# Verification of De-Identification Techniques for Personal Information Using Tree-Based Methods with Shapley Values

**DOI:** 10.3390/jpm12020190

**Published:** 2022-01-31

**Authors:** Junhak Lee, Jinwoo Jeong, Sungji Jung, Jihoon Moon, Seungmin Rho

**Affiliations:** Department of Industrial Security, Chung-Ang University, Seoul 06974, Korea; aa1103@cau.ac.kr (J.L.); jjw0202@cau.ac.kr (J.J.); jjjsj11@cau.ac.kr (S.J.); johnny89@cau.ac.kr (J.M.)

**Keywords:** de-identification, medical data, machine learning, tree-based method, explainable artificial intelligence

## Abstract

With the development of big data and cloud computing technologies, the importance of pseudonym information has grown. However, the tools for verifying whether the de-identification methodology is correctly applied to ensure data confidentiality and usability are insufficient. This paper proposes a verification of de-identification techniques for personal healthcare information by considering data confidentiality and usability. Data are generated and preprocessed by considering the actual statistical data, personal information datasets, and de-identification datasets based on medical data to represent the de-identification technique as a numeric dataset. Five tree-based regression models (i.e., decision tree, random forest, gradient boosting machine, extreme gradient boosting, and light gradient boosting machine) are constructed using the de-identification dataset to effectively discover nonlinear relationships between dependent and independent variables in numerical datasets. Then, the most effective model is selected from personal information data in which pseudonym processing is essential for data utilization. The Shapley additive explanation, an explainable artificial intelligence technique, is applied to the most effective model to establish pseudonym processing policies and machine learning to present a machine-learning process that selects an appropriate de-identification methodology.

## 1. Introduction

With the recent development of big data and cloud computing technologies, numerous data, including personal information, have been generated in digital environments [1]. Hence, many organizations have been striving to efficiently protect their personal information [2,3]. For instance, pseudonym information in South Korea is defined as personal information that cannot be recognized without additional information by processing part of the personal information under an alias, such as deletion or replacement [4], and can be used freely without the need for separate consent [5,6]. However, the failure to conduct the proper procedures for converting personal information into pseudonym information could cause serious privacy problems [5,7,8]. Therefore, an appropriate de-identification process is required to convert personal information into pseudonym information [3,9].

Various studies have been conducted to effectively perform de-identification or develop open-source tools for de-identifying personal information, such as automatic retransmission exchange [10,11]. Nevertheless, few methods evaluate whether de-identified data (including alias processing) can be properly identified. For instance, technology for producing pseudonym information must be transformed for the intended purposes. However, if personal information is too aliased at an unspecified level, although the confidentiality of the data could be increased, the usability could be reduced [12,13]. Hence, excellent de-identification indicates that the data have both confidentiality and usability. Therefore, studies are essential for proposing an appropriate de-identification methodology, considering personal data analysis through independent variables processed in the de-identification methodology in numerical form and dependent variables derived by properly considering the values of data confidentiality and usability [14,15].

Recently, machine-learning techniques have been used for data analysis in a variety of fields, including the education [16,17], energy [18,19], environmental [20,21], medical [22,23,24], and security [25,26,27] fields, to assist professionals in saving time and effort because machine learning can effectively discover nonlinear relationships between dependent and independent variables in numerical datasets. To address classification problems in the medical field, Shailaja et al. [28] presented various machine-learning techniques, such as the support vector machine, naive Bayes classification, *k*-nearest neighbors, and others, to predict various diseases, such as heart disease, breast cancer, diabetes, and thyroid disease. To address regression problems in the medical field, Kwon et al. [29] used the electronic medical records of patients with heart failure and cataract symptoms to develop a recurrent neural-network-based visual analytical tool for obtaining insight into how individual medical codes contribute to risk projections.

However, because the decision-making process within these models is opaque (i.e., a black box), forecasting findings generated by these models cannot be fully trusted and exploited [30]. Therefore, explainable artificial intelligence (XAI) technology has recently garnered increased attention in developing reliable and interpretable forecasting models [31,32]. The XAI technology can change the artificial intelligence (AI) decision-making process, secure reliability, provide stability, and improve model performance. A representative example of XAI technology, the decision tree (DT)-based ensemble learning models with Shapley additive explanation (SHAP) values have achieved excellent performance in various domains and could confirm which independent variables affect the model construction through the Shapley values [33,34]. However, no previous work has employed a de-identification methodology that includes data confidentiality and usability for appropriate personal healthcare data utilization based on XAI techniques.

This paper proposes a verification approach for de-identification methodologies based on the DT-based ensemble learning methods with SHAP values. First, a de-identification dataset is configured for model training. The dataset contains the number of samples and labels of usability and confidentiality for personal information as independent variables, including the final score as a dependent variable via labeling weights considering the confidentiality and usability of the personal information. Several simulation models are constructed to verify the effectiveness of this approach using DT-based methods, exhibiting model interpretability and superior performance in the tabular datasets: DT, random forest (RF), gradient boosting machine (GBM), extreme gradient boosting (XGB), and light GBM (LightGBM). Finally, the performance of the simulation model is compared using a new de-identification dataset, and the interpretability of the best simulation model is presented.

The main contributions of this paper are as follows:

- A generated dataset based on medical data for model training is configured, exhibiting a distribution similar to the actual personal information dataset by considering the actual statistical results for South Korea, including the name, residential area, and age.
- Data preprocessing is performed for the self-de-identification dataset and simulation models using DT-based methods constructed for training various de-identification datasets, and their performance is compared to confirm superior models in terms of privacy.
- An XAI technique is used to interpret the process of model training to present the process for determining an appropriate de-identification methodology that can assist in developing a de-identification methodology or modifying policies in the future.

The remainder of this paper is structured as follows: Section 2 briefly reviews the previous work on data usability and confidentiality. Next, Section 3 summarizes the framework for the proposed approach. Then, Section 4 validates the approach through experiments and interprets the best simulation model using the XAI technique. Finally, Section 5 concludes this paper and suggests directions for future work.

## 2. Related Work

### 2.1. Data Usability

Bloland and MacNeil [35] provided a definitional framework to untangle many of the variables that have previously confounded conversations about the quality of vaccination data. The framework classifies immunization data into three categories: data quality, usability, and utilization. The framework also provides tangible recommendations for a specific set of indicators that could better identify the important qualities of immunization, such as trueness, concurrence, relevancy, completeness, timeliness, integrity, consistency, and utilization. Silsand et al. [36] conducted a formative review of an empirical project in North Norway using a qualitative trailing research approach paired with information infrastructure theory. Parts of the clinical information in the electronic health record (EHR) were formatted as openEHR archetypes in this project to enable automatic data to be reused from the EHR system in a national medical quality registry. They investigated the design problems that arise from organizing clinical information for various uses. As a result, they identified three critical concerns to fix: (1) the need for context when reusing variables, (2) how to verify reusing the correct data, and (3) the difficulties of granulating the variables. The most critical prerequisites for increasing data usability through clinical information structuring were governance and competency. Wait [37] assisted in developing attainable data quality objectives and insight into obtaining reliable results that adequately support the findings when reviewed by others.

Adnan et al. [38] undertook a rigorous systematic review of the literature using the preferred reporting item for systematic reviews and meta-analyses (PRISMA) framework to construct a model to improve the usability of unstructured data and bridge the research gap. The most recent methodologies and solutions for text analytics were thoroughly studied. Concerns regarding the unstructured text data usability and their implications for data preparation for analytics were discovered. The usability enhancement methodology incorporates the definition of usability dimensions for unstructured big data, the discovery of usability determinants, and the development of a relationship between usability dimensions and determinants to produce usability rules. Their proposed model contributes to the usability of unstructured data and simplifies data preparation operations with more valuable data, which ultimately improves the analytical process. They also discovered unstructured big data usability difficulties for the analytical process to bridge the identified gap [39]. The usability enhancement approach for unstructured big data has been presented to improve the subjective and objective efficacy of unstructured big data for data preparation and manipulation operations. Furthermore, idea mapping was a crucial component for improving the usability of unstructured big data in the suggested model with usability principles. These principles bridged the usability gap between the availability of data and their usefulness for the intended purpose. The proposed study methodology could help improve unstructured big data analytics efficiency.

### 2.2. Data Confidentiality

Javid et al. [40] underlined the difficulties of cyber security and data privacy and developing solutions in adopting Industry 4.0 in the healthcare industry. For example, a reduction in the attack surface is required to seamlessly integrate complex computational algorithms, such as those used in cryptography. This issue can be solved by employing Cloudlet technology, which employs virtual machines near the mobile device to assist with preliminary big data analysis for wireless body area networks. Furthermore, the authors suggested several possibilities from Cloudlet technology for future research, such as supported remote robotic surgery. Domingo-Ferrer et al. [41] used the utility in the traditional sense of retaining the statistical properties of the original data. They specifically used the unified perspective of anonymization of the permutation model to create constrained confidentiality metrics for microdata anonymization based on the relative quantities of permutations by the different attributes of a dataset. They presented experimental results demonstrating that their proposed metrics produce outcomes consistent with intuition for several anonymization approaches in the literature, including privacy models and statistical disclosure control methods based on the noise and generated data. 

Yuan et al. [42] proposed a comprehensive scheme that simultaneously achieves data privacy protection, data dynamics, and batch auditing in a public cloud storage environment. This scheme can safeguard data blocks during audits and effectively support data dynamics. Furthermore, third-party auditors can do batch audits for many users. Finally, the security analysis demonstrated that the scheme is risk-free. Gai et al. [43] focused on privacy and proposed a revolutionary data encryption strategy called the dynamic data encryption strategy (D2ES). They recommended selective data encryption and using privacy categorization methods under time restrictions. This approach was designed to maximize the extent of privacy protection by employing a selective encryption mechanism within the execution time constraints. In their experiments, the performance of D2ES was examined, verifying the privacy enhancement. Bakir [44] developed a model that includes three key characteristics: data confidentiality, integrity, and consistency of information security for massive datasets. A more practical and adaptable structure was realized with the single labeling model for all database operations (reading, writing, updating, and deleting) on actual data. Therefore, all processes were given with the three key features. The outcomes of the proposed single-label model were compared to the application and experimental investigation that the author conducted, and the findings are encouraging for further research.

However, no previous work on personal health information has been conducted that assesses whether alias processing was successfully performed using a de-identification methodology that considers data confidentiality and usability for appropriate data utilization. In addition, which variables had an effect when verifying data de-identification were not easily determined in the existing studies.

## 3. Materials and Methods

This paper generates a de-identification dataset focusing on EHRs, which were actively conducted for an experimental evaluation of de-identification tools. First, personal information was collected from the statistical results of government institutions in South Korea, such as the Korean Statistical Information Service (KOSIS) and the Ministry of Health and Welfare (MOHW). Various de-identification methodologies were applied to determine the labels for the usability and confidentiality of personal information.

**Figure 1:**
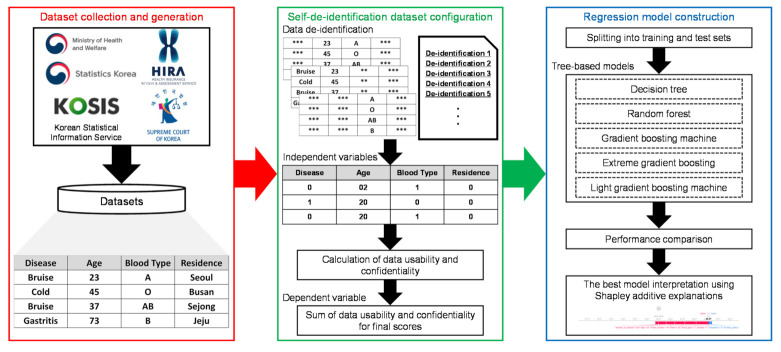
Overall architecture of the proposed approach. An asterisk (*) indicates a de-identified character from personal information.

The total number of samples and labels applied to each de-identification dataset was configured into one tuple for independent variables in the sample dataset. Figure 1 illustrates the overall architecture of the proposed approach. In the case of data confidentiality in this paper, the masking tape technique, the most commonly used data de-identification technique, was used to cover part of the characters to prevent privacy exposure [45,46]. The de-identification methodology was treated as a number and used as the value of the independent variables.

Afterward, the de-identification methodology was applied to the sample dataset to calculate the confidentiality and usability of the labeling data to determine scores for the de-identification methodology. The DT-based methods that demonstrate excellent performance and model explanatory properties for AI techniques were applied to construct a model that predicts the value of the dependent variable by learning the independent variable expressed numerically.

Thus, a de-identification dataset was generated and guided by collecting personal information, creating itemized rules, and applying the de-identification methodology. In addition, this approach can be employed for medical data and application and verification in other areas by considering various items.

### 3.1. Data Generation

The data considered in this paper consist of personal information that, without de-identification processing, cannot be used directly under the current privacy law in South Korea. Therefore, virtual data, such as the actual statistical percentage from the KOSIS, were generated by referring to the statistical results of the Family Relationship System, the Population and Housing Census, and public medical data. The virtual data area was set as medical data, and the items of the virtual data consist of the name, age, phone number, residential area, illness, blood type, and smoking status.

#### 3.1.1. Name

**Figure 2:**
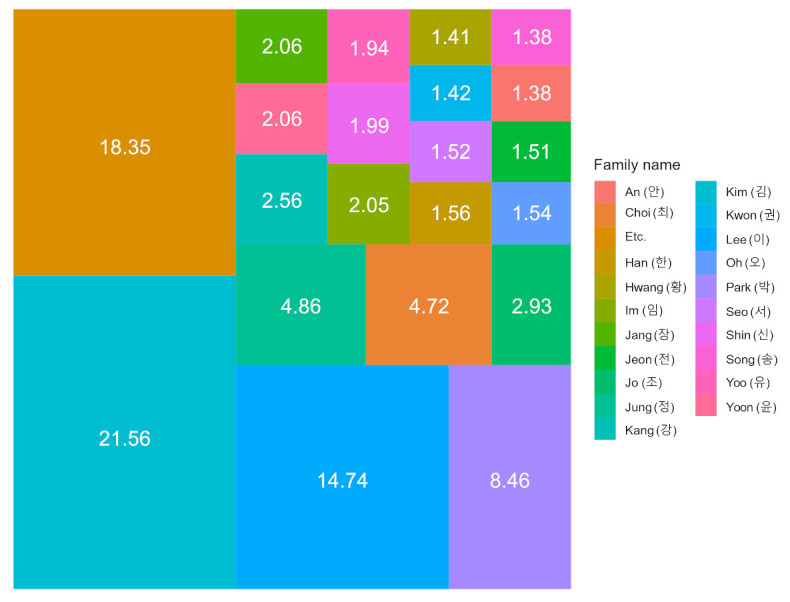
TreeMap of leading family names and their percentages.

Various virtual names were generated by considering the family and given names. Both names were generated based on the statistical results from the Family Relationship System [47,48]. According to the distribution of family names in South Korea, the leading family names and their percentages are as follows: Kim (21.56%), Lee (14.74%), Park (8.46%), Jung (4.86%), Choi (4.72%), Jo (2.93%), Kang (2.56%), Yoon (2.06%), Jang (2.06%), Im (2.05%), Shin (1.99%), Yoo (1.94%), Han (1.56%), Oh (1.54%), Seo (1.52%), Jeon (1.51%), Kwon (1.42%), Hwang (1.41%), An (1.38%), Song (1.38%), and 98 other family names (18.35%). Figure 2 presents a TreeMap of the leading family names and their percentages.

#### 3.1.2. Age

**Figure 3:**
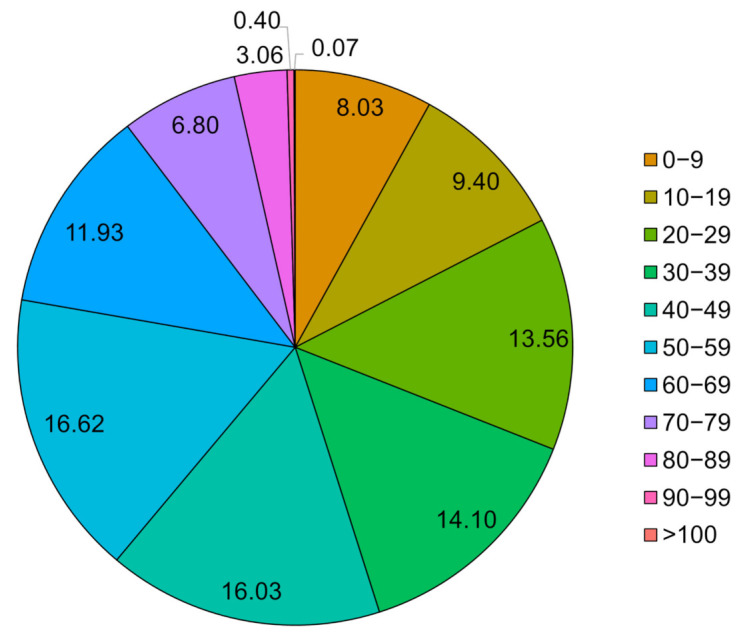
Pie chart of the percentage of each age group in the South Korean population.

Age values were generated by considering the statistical results from the Population and Housing Census. Although the Population and Housing Census presents five age intervals (i.e., 19–29, 30–39, 40–49, 50–59, and 60–75), 11 intervals were determined to be appropriate for dataset configuration to effectively reflect the characteristics of the age group in the medical data. Thus, 10-year age intervals between 0 and more than 100 were set by calculating and reflecting on the population of South Korea in 2015 (Figure 3). The percentages for each age interval are as follows [49]: 0–9 (8.03%), 10–19 (9.40%), 20–29 (13.56%), 30–39 (14.10%), 40–49 (16.03%), 50–59 (16.62%), 60–69 (11.93%), 70–79 (6.80%), 80–89 (3.06%), 90–99 (0.4%), and >100 (0.07%).

#### 3.1.3. Phone Number

For phone numbers, because no rules or overlapping values apply for numbering in South Korea, the statistical results for their distribution do not exist. Hence, all values except for 010 were randomly generated with the first three numbers in common.

#### 3.1.4. Residential Area

The Population and Housing Census was used to describe the urban residential area dataset [50]. The population distribution was considered for all provinces and metropolitan cities in South Korea, as listed in Table 1.

#### 3.1.5. Illness

The five considered illnesses were randomly distributed in EHRs: colds, headaches, gastritis, body aches, and bruises without any other statistical distribution. Unlike other characteristics, as this data item is directly used after de-identification processing, it does not have a specific generation probability; therefore, it can have a completely random distribution.

#### 3.1.6. Blood Type

According to the Korean Red Cross Blood Information Statistics [51], Rh^+^ comprises 99.7% of blood types in the South Korean population. The distribution of each blood type is A (34%), B (26.6%), O (27.5%), and AB (11.4%). Hence, only Rh^+^ was considered because Rh^−^ does not cause any significant difference in proportion.

#### 3.1.7. Smoking Rate

According to the MOHW and the Korean Centers for Disease Control and Prevention, the smoking rate in South Korea is 21.5% (men: 35.7%, women: 6.7%) [52]. A virtual dataset for the smoking rate was generated considering the actual statistical distribution.

### 3.2. Data Preprocessing

As mentioned, a de-identification dataset was configured for the independent variables. A dependent variable must be provided for model training based on the de-identification dataset with masking. Hence, a masking methodology to apply de-identification was proposed for the specified items via rules. The de-identification methodology for each sample and characteristic was expressed numerically, and then these values were adopted as each tuple of the dependent variable.

Good de-identification data are defined as having high confidentiality and usability to estimate the dependent variables, called score values, corresponding to the exact data before generating the masking methodology. Data confidentiality represents the probability that an individual can be specified within a de-identification dataset. Within a de-identified dataset, the specific personal information sought (a line from the de-identification dataset) may be specified, meaning that de-identification processing has not been properly performed in terms of privacy.

Data usability refers to how de-identified data can be used as pseudonym information in the future. If the de-identification is excessive, the value may significantly decrease when using the data as pseudonym information later. Thus, “well de-identified” means that data confidentiality and usability are properly considered. Data confidentiality and usability tend to be inversely proportional to the degree of application of the de-identification methodology; therefore, they should be unidentified to protect personal information while finding an appropriate intermediate form to make information meaningful when used. Data confidentiality and usability were incorporated into the evaluation to reflect the computation of the score values corresponding to the correct answer data for tree-based model training.

Data confidentiality is expressed as a percentage by dividing the number of groups deduplicated after de-identification by the number of original data in a de-identification dataset, multiplied by 100, as presented in Equation (1). The term “number of deduplicated groups” means the number of data remaining when one completely redundant column is left and excluded (smaller values indicate that the individual is less likely to be identified in that dataset):

Data confidentiality = (no. of deduplicated groups after de-identification/no. of original data) × 100.(1)

Data usability is calculated using labeled values via Equation (2). Labeling means converting the de-identification methodology applied to each characteristic of the feature data into integer values for model training. When performing this labeling, the labeling value is determined to consider the data usability. Subsequently, the characteristics of the feature data are multiplied by a single digit and a decimal digit for each labeled value, and then all these values are added to calculate the data usability. The labeling rules applied to each data characteristic are introduced below:

Data usability = ∑ (decimal digit × single digit of labeled value).(2)

#### 3.2.1. Name

**Figure 4:**
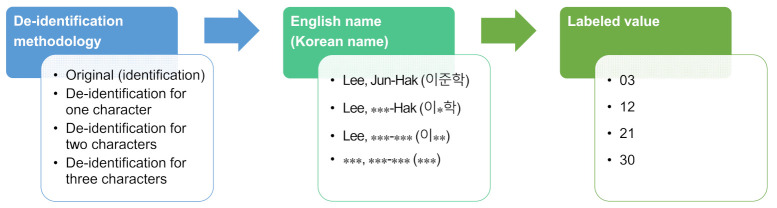
Labeling rules for the family name. An asterisk (*) indicates a de-identified character from personal information.

For the names, three-character names (Korean characters), accounting for most of the names in South Korea, were labeled 03, 12, 21, and 30. For two-character names, which character was masked was not considered, as it was concluded that there was no distinction because the data usability does not differ depending on which character was masked. Therefore, the same labeling value was reflected in the input data regardless of the value or location if the characters were masked. Figure 4 presents the final set of rules considered in this paper.

#### 3.2.2. Age

The age category, like the name category, was set to **% from 0 to 99 years of age. The content of the age data was categorized as 0–9, and, unlike decimals, the number of digits is not significant in terms of data usability; therefore, the number of cases in which only the decimals were masked was excluded, as listed in Table 2. If the original is masked with nothing, only the one is masked, or both numbers are masked, the labeled values are 02, 11, and 20, respectively.

Specifically, for the labeled value, the first digit is the number of masked digits, and the next digit is the number of digits exposed without masking. In the original data, both digits are exposed; thus, the first digit in the value is 0, and the last digit is 2, resulting in 02. For the de-identification of one digit, one digit is exposed, and one is masked, and therefore the first and second digits are both 1, and the resulting labeled value is 11. For the de-identification of two digits, as both are masked, there are no exposed digits; thus, the first digit is labeled 2, and the last digit is labeled 0, resulting in 20.

#### 3.2.3. Phone Number

Phone numbers have properties similar to unique identification numbers in that they are generated randomly and without duplication, and knowing the value within the dataset can specify the data. Thus, more released digits result in a less distinctive number of groups, and the more effective use of phone numbers results in a more significant number of digits to be identified regarding the data usability value.

Concerning the data usability, the effect on the number of unique groups was more significant than the value of the phone numbers, which needed to be adjusted. Therefore, all four digits were masked, front and back, and one out of eight digits was masked. Previously, the South Korean phone number system used 011, 017, and so on as the first digits, a system that contained a region or other characteristics. However, South Koreans can now choose any number without cost. In this regard, 08, 17, and 80 were all labeled values, as listed in Table 3.

#### 3.2.4. Other

Additionally, four blood types, 17 addresses, five illnesses, and two smoking conditions were divided into the training data. We judged that masking only a part of the information, such as one or two letters, is meaningless. Even if part of the information is masked, the information is eventually categorized into groups according to the given number unless all the information is masked, and thus this method distinguishes whether to mask the information, as presented in Table 4. The characteristics corresponding to the categorized data are blood type, address, illness, and smoking status.

#### 3.2.5. Data Usability

For dataset configuration, data generation was performed considering only seven items: name, age, phone number, blood type, address, illness, and smoking status. However, using a simple ruleset makes it difficult to demonstrate the need for AI techniques and does not guarantee a fundamental purpose. Therefore, a novel value called the data usability was automatically formulated and calculated for all labeling values as listed in Table 5. The data usability was calculated using this determined data labeling rule. As mentioned, the values were obtained by adding digits from each category. Each digit was multiplied and added in the categories of name, age, and telephone number. Each digit was simply added in other cases of blood type, address, illness, and smoking status. Thus, data de-identification methods were obtained that consider the data usability.

#### 3.2.6. Data Confidentiality

As mentioned, data confidentiality items were created to reflect the effect of privacy through de-identification processing and data usability. A smaller number of distinct groups within a de-identification dataset makes specifying individuals less likely. However, the amount of data for each de-identification dataset could vary greatly; therefore, the number of identical groups for the number of original data groups was calculated as a percentage. The values were calibrated by scoring them in intervals. As reflecting the percentage in the final score value calculation can lead to abnormally high data confidentiality scores compared to the calculated data usability, the data confidentiality values were divided by intervals according to the percentage value, and the appropriate data confidentiality values were set. For data usability, the maximum value of the scores reflected by the interval is 15, and the minimum value was 0.

Therefore, the data confidentiality was adjusted by three to maintain the same interval, as presented in Table 6. The intervals were initially divided by 20%, but if the number of groups after de-identification and the number of groups of original data were the same, the de-identification was not properly performed, and then one point was added to reflect the interval between 99 and 100.

#### 3.2.7. Final Score

When data preprocessing, including data labeling, usability, and confidentiality for all items, was completed, a final score value (sum of data confidentiality and usability) was calculated to determine the dependent variable. Consequently, several simulation models using tree-based methods were constructed with the name, age, phone number, residential area, illness, blood type, smoking status, and subsequent labeling values as independent variables and the final score value as the dependent variable.

### 3.3. Model Construction

Tree-based methods are the most robust supervised learning techniques for classification and regression [53,54,55]. These methods construct forecasting models that provide satisfactory performance, high accuracy, and easy-to-understand interpretation [53]. Unlike linear models, tree-based methods can fit nonlinear relationships and cover many kinds of problems in machine learning [55,56]. Five tree-based methods were used: DT, RF, GBM, XGB, and LightGBM.

The DT [53,57] exhibits two features: tree structure modeling and rule-based search. First, the DT splits the dataset multiple times according to specific boundary points in the variable. Splitting generates different subsets of the dataset. The final and intermediate subsets are terminal or leaf nodes and internal or split nodes, respectively. Second, because the DT could represent a classification or regression process based on an inference rule, it is easier to understand compared with other AI techniques. However, the DT recognizes continuous variables as noncontinuous variables; thus, it could derive a significant prediction error near the boundary point [57]. Therefore, ensemble learning methods using multiple DTs, such as bagging and boosting, have been developed to address this issue. The bagging method [58] generates several weak learners (e.g., DTs) using the dataset extracted from random sampling and then aggregates the results by considering voting (classification) or averaging (regression), thus, reducing the variance and noise. The most popular bagging method, RF [57,58], has two primary hyperparameters: the number of trees and features. The former parameter determines the number of randomly generated DTs, whereas the latter specifies the number of independent variables reflected in generating the DT. A larger number of features results in a more similar generated tree form. Moreover, fewer features reduce the overfitting due to different DT forms.

The GBM [53,59], a typical boosting method that trains iteratively by estimating using one weak learner (e.g., DT) and passing the remaining residuals back to other weak learners, uses the stochastic gradient descent algorithm to minimize the loss function. However, building the GBM is time-consuming due to repetitive training. Chen and Guestrin [60] developed XGB to address the problem by enabling parallel processing that classifies each bucket via the split-finding process. The XGB could be selected as two opportunities for a weak learner (i.e., tree and linear functions). The tree function that uses the regression tree as a weak learner was considered because this paper proposes the prediction performance comparison of tree-based methods. The LightGBM [61,62] is a boosting-based algorithm with faster speed and higher forecasting accuracy compared with other boosting and bagging algorithms. It is based on a gradient boosting DT with gradient-based one-sided sampling and exclusive feature bundling technologies [62]. Unlike the level method, the leaf method creates complex models to achieve higher accuracy. Therefore, it is useful for time series forecasting, and due to the gradient boosting DT and leaf method, LightGBM has reduced memory usage and a faster training speed. In addition, the LightGBM contains many hyperparameters closely related to the forecasting accuracy, such as the learning rate, number of iterations, and number of leaves.

## 4. Results

### 4.1. Experimental Design

Training and testing of the machine-learning model were conducted in the following environment: an LG Electronics laptop with an Intel(R) core (TM) i3-6100U CPU at 2.30 GHz with RAM 8 GB with Python 3.8.8 (Python Software Foundation, Delaware, MD, USA) and scikit-learn 0.23.2. First, it was necessary to perform data preprocessing before tree-based model training. After reading the original data using Pandas, the independent variables (sample number, phone number, age, etc.) and the dependent variable (final score) were configured. Then, these two datasets were divided into the training (80%) and testing (20%) sets.

### 4.2. Performance Indicator

Two popular metrics were employed, the root mean square error (RMSE) and *R*-squared (*R*^2^), to compare the prediction performance of the tree-based models using Equations (3)–(5). The RMSE value is the sum of the squares of the predicted value (*P_t_*) and the actual value (*A_t_*) divided by the number of samples (*n*). This metric is equivalent to the concept of the sum of the square error for the analysis of variance, and the sum of square error values divided by the number of data is the mean square error (MSE). Thus, the root of this value is RMSE. Therefore, the learned linear machine-learning model represents the difference between the predicted and actual values; a smaller RMSE value indicates that better actual data are predicted:

MSE = ∑ (*P_t_* − *A_t_*)^2^/*n*, (3)

RMSE = √MSE.(4)

Furthermore, *R*^2^ measures the fit of the regression models. After a regression model has been proposed, *R*^2^ is one of the goodness-of-fit statistical methods to determine how well the model fits the actual value. The *R*^2^ value is between 0 and 1, and a higher value indicates a better regression model. The value of *R*^2^ can also be interpreted as the variance of the predicted value relative to the variance of the actual value, indicating how strongly the regression model correlates with the actual value:

*R*^2^ = 1 − (∑ (*P_t_* − *A_t_*)^2^/(∑ (*A_t_* − *A_avg_*)^2^), (5)

where *A_avg_* is the mean of the actual values.

### 4.3. Hyperparameter Tuning

Most DT-based methods contain hyperparameters that significantly affect the model performance. The optimal hyperparameters for each tree-based method were determined through five-fold cross-validation in the training set. Table 7 lists the hyperparameters for the tree-based methods and their ranges.

### 4.4. Performance Comparison

The most appropriate tree-based model was selected through optimal hyperparameter tuning. Table 8 displays the five methods, the selected hyperparameter values with the lowest RMSE and highest *R*^2^ values for each algorithm, and the training time. With the lowest RMSE value, LightGBM scored the highest at 0.986, and thus it was chosen as the best algorithm model for de-identification methodology.

The LightGBM model has the best *R*^2^ and RMSE values in the testing set. This model’s learning and prediction times and memory usage are less than the other tree-based methods. Through the application of AI and XAI (explained in the Model Interpretation section later), this model also has great strength in that it can perform analyses on machine-learning models, which can be used to further understand machine learning and predictive performance.

### 4.5. Model Interpretation

Recently, with the legal basis for data utilization, AI-based models have become more sophisticated and developed. However, when AI achieves a result, it is challenging to track the validity of the process and the rationale for achieving the result. Even developers who have participated in developing AI models cannot understand why these results were derived and on what basis, and they cannot rely entirely on black-box AI. Thus, XAI is emerging, which can explain and present decisions or answers derived from AI in a way that people can understand.

The SHAP is a method for analyzing the influence of input variables by calculating the SHAP value for each input variable. The SHAP value is the Shapley value for the machine-learning model’s conditionally anticipated value function. When comparing the results for the input instance with the expected value for the model without a given input variable, the relevance of the Shapley value indicates the input variable relevance. Consequently, examining the model based on SHAP can interpret which input variables are important and study the change in the output as the input variable values vary.

**Figure 5:**
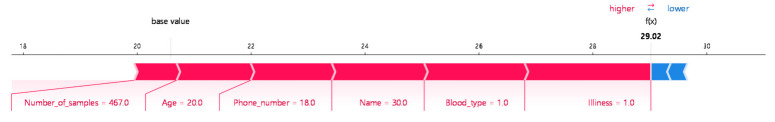
Sample Shapley additive explanation force plot for final score prediction.

**Figure 6:**
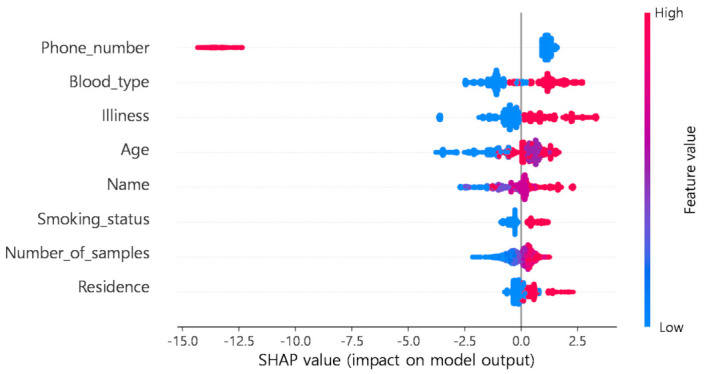
Summary plot of selected Shapley additive explanation (SHAP) values.

The SHAP determines the Shapley value of the learning model using the conditional expected value function and includes the kernel SHAP, which applies to all machine-learning models, deep SHAP, which applies to neural network models, and tree SHAP, which applies to the DT-based ensemble-learning model. This paper employs the tree SHAP to interpret the LightGBM process [33,34]. Thus, how much influence each attribute has and how each property affects the LightGBM model were analyzed as illustrated in Figure 5 and Figure 6.

First, the SHAP value demonstrates how each attribute visually affects the LightGBM model trained earlier. Above is a summary of the SHAP values for all variables. Red and blue variables indicate positive and negative influences on the target, respectively. The interpretations of the variables are as follows:

- Phone number: The lower the value, the higher the expected score.
- Blood type: The higher the value, the higher the expected score.
- Illness: The higher the value, the higher the expected score.
- Age: The higher the value, the higher the expected score.
- Name: The higher the value, the higher the expected score.
- Smoking status: The higher the value, the higher the expected score.
- Number of samples: The higher the value, the higher the expected score.
- Residence: The higher the value, the higher the expected score.

Before creating the input data, labeling was performed on each property, and the larger the labeled value, the less useful the data are overall, which lowers the data usability value in the final score. In contrast, because of the larger labeled values, the data are more confidential, and the number of groups is smaller, increasing the data confidentiality values in the final score.

**Figure 7:**
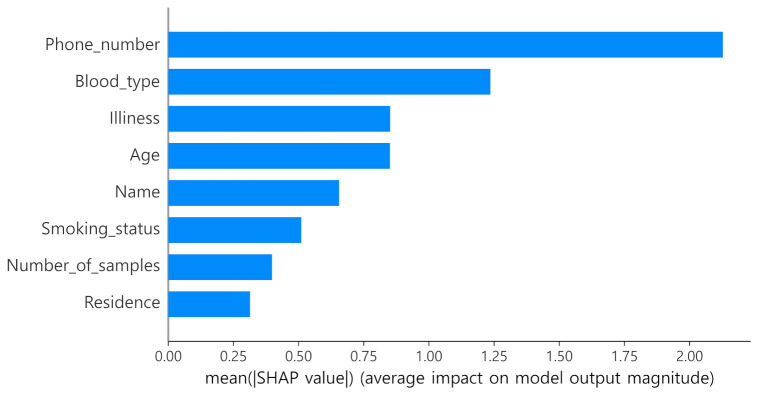
Feature importance based on Shapley additive explanation (SHAP) values.

The visualization results in Figure 7 indicate that the higher the value of a variable for most properties, the higher the expected score, indicating that the degree of de-identification had a greater effect on determining the final score values. Regarding the importance of each attribute, the phone number exhibits the greatest feature importance. Perhaps because phone numbers appear as random numbers, they rarely belong to the same group, which significantly affects the scores.

### 4.6. Discussion

No matter how a LightGBM model could verify a de-identification technique accurately and interpretably, it is challenging to adopt it in the industry if it does not yield a better prediction performance than other famous regression models. According to [22], multiple linear regression (MLR), Gaussian process regression, support vector regression (SVR), and deep neural network (DNN) are considered as famous regression models in the medical field along with DT and RF. The performance of the LightGBM model was compared with that of the MLR, SVR, and DNN models to confirm the applicability to the medical field. The Gaussian process regression model was not considered a benchmark model in this study because of the memory limitation issue in the computer environment. Grid search via five-fold cross-validation was used to optimize several hyperparameters of the SVR and DNN models.

**Figure 8:**
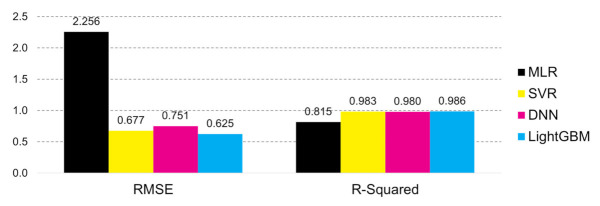
Performance comparison of four famous regression models in the medical field. Multiple linear regression (MLR); support vector regression (SVR); deep neural network (DNN); and Light gradient boosting machine (LightGBM).

Figure 8 presents the values of some performance indicators (RMSE and *R*^2^) in Table 8. The LightGBM model performed better than the MLR, SVR, and DNN models on the verification of data de-identification. Hence, because the LightGBM model presents model interpretability and excellent performance, it could be sufficiently applicable in the medical field.

The proposed scheme has the advantage of checking whether variables have influence while delivering the best performance for the verification of data de-identification. Furthermore, by numerically describing how well data confidentiality and usability are handled through labeling, the scheme may verify previously unknown data de-identification procedures. In particular, the proposed technique derives a numeric value in consideration of the confidentiality and usability of labeling, and when this numeric value is used, excellent learning results are derived.

Although the proposed model has outstanding advantages, several limitations exist. Other state-of-the-art methods may outperform the proposed model in the distant future. However, if better ways of expressing data confidentiality and usability emerge, more suitable AI models may emerge. Although SHAP increases the decision-making reliability, the results and their reliability can be a problem if the data used to make decisions are unreliable. In addition, even with XAI, it is impossible to interpret all logic trees used in the machine-learning model and track associations. In other words, it means that XAI must admit that there is a limit to fully explaining the causal relationship of learning. Nevertheless, the SHAP method considers the likelihood that variables influence one other and can quantify the negative influence. Hence, despite its disadvantage of being slow, it is clear that it assesses influence more precisely than the variable importance method.

Many machine-learning algorithms have been developed for laptops or desktop computers. As machine-learning algorithms have issues with performance, memory capacity, and algorithm size [63,64,65], their use on mobile devices, such as smartphones and tablets, is limited. The XGBoost and LightGBM algorithms emerged to alleviate the GBM’s excessive memory usage, and it is a significant challenge in machine learning to make the methods lightweight. Further study on simplifying the algorithm model suggested in this paper must be implemented in mobile devices in the future because the algorithm’s weight reduction allows flexible use in many devices, which is directly related to user convenience.

## 5. Conclusions

Recently, as the legal grounds for using pseudonym information have been established in various countries, research has been conducted regarding pseudonym information. However, this requires the proper processing of aliases (de-identification). This study proposed an AI framework to determine whether alias processing has been properly performed through a de-identification methodology. As it yields scores that consider data confidentiality and data usability for appropriate data utilization, data users can observe a great effect in verifying de-identification methodologies.

Furthermore, the best methodology can be proposed in reverse when companies and others process aliases. After applying various de-identification methodologies using sample counts and privacy items, the scores for each methodology can be compared to determine the de-identification methodology with the highest score value. This method allows data users to determine appropriate de-identification methodologies that are superior for data confidentiality and data usability as objective indicators.

A limitation of this study is that the original data used to design the AI framework were not actual data but generated virtual data. These data were used because using factual personal information is legally problematic, and it is meaningless to de-identify the data again because pseudonym information has already been de-identified. Thus, the study compromised by generating various items in the original dataset while considering the actual statistical distribution.

In addition, whether the weight values for data confidentiality and usability scores represent the confidentiality and usability of the data is another limitation of this study. When labeling and calculating the score values of the data usability, a higher weight should be assigned to meaningful data, and the definition of meaningful should be objective and specific. In the future, more objective indicators will be continuously assessed to legally use personal information from the real world to generate original data with more rigorous consideration of the statistical distribution and to label and calculate data confidentiality and usability scores.

## Figures and Tables

**Table 1:** Province and metropolitan city information for South Korea.

Name	Provincial Level	Percentage
Seoul	Special city	18.5
Busan	Metropolitan city	6.5
Daegu	Metropolitan city	4.7
Incheon	Metropolitan city	5.7
Gwangju	Metropolitan city	2.9
Daejeon	Metropolitan city	2.9
Ulsan	Metropolitan city	2.2
Sejong	Special self-governing city	0.7
North Chungcheong	Province	3.1
South Chungcheong	Province	4.2
Gangwon	Province	2.9
Gyeonggi	Province	26.1
North Gyeongsang	Province	5.1
South Gyeongsang	Province	6.4
North Jeolla	Province	3.5
South Jeolla	Province	3.5
Jeju	Special self-governing province	1.3

**Table 2:** Labeling rule for age. An asterisk (*) indicates a de-identified character from personal information.

De-Identification Methodology	Example	Labeled Value
Original (identification)	12, 25	02
De-identification for one character	1 *, 2 *	11
De-identification for two characters	**, **	20

**Table 3:** Labeling rule for phone number. An asterisk (*) indicates a de-identified character from personal information.

De-Identification Methodology	Example	Labeled Value
Original (identification)	010-1234-5678	08
De-identification for one character	010-1***-****	17
De-identification for all characters	***-****-****	80

**Table 4:** Labeling rule for other items. An asterisk (*) indicates a de-identified character from personal information.

De-Identification Methodology	Example	Labeled Value
Original (identification)	bruise	1
De-identification for all characters	*	0

**Table 5:** Example of calculation for data usability. An asterisk (*) indicates a de-identified character from personal information.

Independent Variables	Example	Labeling Rule
Name	Lee, ***-Hak	12
Age	2 *	11
Phone number	010-7 ***-****	17
Blood type	A	1
Address	Seoul	1
Name of illness	*	0
Smoking status	X	1
Data usability	(1 × 2) + (1 × 1) + (1 × 7) + (1) + (1) + (0) + (1)	13

**Table 6:** Example of the calculation of data confidentiality.

Percentage	Confidentiality Value
0–20%	15
21–40%	12
41–60%	9
61–80%	6
81–99%	3
99–100%	1
100%	0

**Table 7:** Hyperparameters and ranges of decision-tree-based methods.

Method	Hyperparameters and Ranges
DT	min_samples_split (minimum number of samples required to split an internal node): 2, 7 min_samples_leaf (minimum number of samples required to be a leaf node): 2, 7 max_depth (maximum depth of the tree): 4, 8, 10, 11, 12, 15
RF	n_estimators (number of trees in the forest): 10, 20, 30, 100, 200, 300, 400 max_depth: 3, 15 min_samples_leaf: 10
GBM	n_estimators: 10, 20, 30, 100, 200, 300, 400, 500, 600, 700 learning_rate (learning rate): 0.02, 0.1
XGBoost	objective (learning task and corresponding learning objective): reg:squarederror (regression with squared loss) learning_rate = 0.01, 0.05, 0.1 n_estimators: 20, 100, 200, 300, 400, 500 min_child_weight (minimum sum of instance weight needed in a leaf): 5 max_depth: 3, 5, 6, 7, 10
LightGBM	num_estimators: 20, 100, 200, 400, 600, 700, 800, 900, 1000, 1100 learning_rate: 0.01, 0.05 max_depth: 3, 7, 15 min_child_samples (minimum number of samples needed in a leaf): 5

DT, decision tree; RF, random forest; GBM, gradient boosting machine; XGBoost, extreme gradient boosting; and LightGBM, light gradient boosting machine.

**Table 8:** Root Mean Square Error (RMSE) and R-square comparison.

Methods	Hyperparameter Values	RMSE	*R* ^2^	Time (s)
DT	min_samples_split: 7 min_samples_leaf: 7 max_depth: 15	0.666	0.985	0.926
RF	n_estimators: 250 max_depth: 15 min_samples_leaf: 10	0.633	0.982	15.066
GBM	n_estimators: 100 learning_rate: 0.1	1.079	0.924	191.734
XGBoost	random_state: 0 objective: reg:squarederror learning_rate: 0.01 n_estimators: 500 min_child_weight: 10 max_depth: 8	0.642	0.986	65.578
LightGBM	num_estimators: 500 learning_rate: 0.05 max_depth: 15 min_child_samples: 10	0.625	0.986	21.336

DT, decision tree; RF, random forest; GBM, gradient boosting machine; XGBoost, extreme gradient boosting; and LightGBM, light gradient boosting machine.

## Data Availability

Not applicable.
